# EDEM1 Drives Misfolded Protein Degradation via ERAD and Exploits ER-Phagy as Back-Up Mechanism When ERAD Is Impaired

**DOI:** 10.3390/ijms21103468

**Published:** 2020-05-14

**Authors:** Marioara Chiritoiu, Gabriela N. Chiritoiu, Cristian V. A. Munteanu, Florin Pastrama, N. Erwin Ivessa, Stefana M. Petrescu

**Affiliations:** 1 Department of Molecular Cell Biology, Institute of Biochemistry, Splaiul Independentei 296, 060031 Bucharest 17, Romania; mari.chiritoiu@biochim.ro (M.C.); gabi.chiritoiu@biochim.ro (G.N.C.); 2 Department of Bioinformatics & Structural Biochemistry, Institute of Biochemistry, Splaiul Independentei 296, 060031 Bucharest 17, Romania; cristian.v.a.munteanu@gmail.com (C.V.A.M.); florinpastrama@yahoo.com (F.P.); 3 Center for Medical Biochemistry, Max Perutz Labs, Medical University of Vienna, A-1030 Vienna, Austria; n-erwin.ivessa@meduniwien.ac.at

**Keywords:** EDEM1, ERAD, ER-phagy, autophagy, protein quality control, mass spectrometry, protein degradation, endoplasmic reticulum, intrinsically disordered region, EDEM1 interaction network

## Abstract

Endoplasmic reticulum (ER)-associated degradation (ERAD) is the main mechanism of targeting ER proteins for degradation to maintain homeostasis, and perturbations of ERAD lead to pathological conditions. ER-degradation enhancing α-mannosidase-like (EDEM1) was proposed to extract terminally misfolded proteins from the calnexin folding cycle and target them for degradation by ERAD. Here, using mass-spectrometry and biochemical methods, we show that EDEM1 is found in auto-regulatory complexes with ERAD components. Moreover, the N-terminal disordered region of EDEM1 mediates protein–protein interaction with misfolded proteins, whilst the absence of this domain significantly impairs their degradation. We also determined that overexpression of EDEM1 can induce degradation, even when proteasomal activity is severely impaired, by promoting the formation of aggregates, which can be further degraded by autophagy. Therefore, we propose that EDEM1 maintains ER homeostasis and mediates ERAD client degradation via autophagy when either dislocation or proteasomal degradation are impaired.

## 1. Introduction

Protein folding and quality control at the endoplasmic reticulum (ER) of a cell is a highly regulated process that ensures proper cell functioning. Despite specialised mechanisms, a considerable fraction of newly synthesised polypeptides fail to attain their native conformation and need to be targeted for degradation [1]. This process involving the recognition, retro-translocation, and proteolysis in the cytosol by the ubiquitin-proteasome system is generally termed endoplasmic reticulum-associated protein degradation (ERAD) [2]. While the last step is relatively well documented, the recognition and retro-translocation of ERAD substrates are currently under intense scrutiny. Following the model of protein folding assisted by lectin chaperones, such as calnexin and calreticulin, it has been proposed that the mannose trimming of N-glycans exposed on misfolded polypeptides is a signal for degradation via ERAD [3,4,5]. Mannose processing proteins such as ER mannosidase I and EDEM (ER-degradation enhancing α-mannosidase-like) family of proteins have been shown to catalyse mannose trimming for glycans exposed on partially folded or misfolded glycoproteins, therefore accelerating their degradation [6,7,8].

ERAD is a dynamic process that has been shown to involve both ER luminal (lectins: OS-9 and XTP3-B; disulfide isomerases: ERdj5; or co-chaperones: ERdj3 and ERdj4), as well as ER-membrane proteins (adaptor protein-SEL1L, E3 ubiquitin ligase-HRD1), which work in concert to select and dislocate misfolded polypeptides from the ER to the cytosol for proteasomal degradation [9]. Many other proteins have been proposed to function as part of ERAD, and different clusters mainly concentrated around E3-ubiquitin ligases could function independently in protein degradation [10]. To date, EDEM proteins have been functionally associated with the HRD1-nucleated complex and have been shown to associate with the adaptor protein of HRD1: SEL1L [11,12,13].

Assigning a role for the mammalian homologues of the yeast Htm1, EDEM1 and the other two members of the EDEM family in ERAD has been highly controversial. EDEM1 has been described as an ER resident protein whose expression is under the control of the unfolded protein response (UPR) and extracts misfolded polypeptides from the calnexin cycle as a first step of ERAD [3,14]. Its structural homology with the ER mannosidase has led to the hypothesis that the mannosidase-like domain recognises high-mannose N-glycans attached to proteins [4,5]. However, several reports have suggested that EDEM1 associates with ERAD substrates independently of its mannosidase domain [15,16,17,18]. We showed that besides the structured mannosidase domain, this protein has an intrinsically disordered region (IDR) that has been predicted to facilitate protein–protein interactions [19]. In addition, more recent reports have stated that recognition of the glycosylated substrates by EDEMs is favoured by their unfolded status, thus supporting the idea of the protein–protein interaction of EDEM1 with misfolded proteins [20].

EDEM1 is also associated with processes such as the degradation of orphan oligomeric subunits, the formation of aberrant oligomeric structures [21,22,23,24] and the formation of LC3-positive structures after virus infection, required for virus replication [25,26,27] or, in other cases, for its own turnover [28,29]. All these suggest that EDEM1 might also function in concert with other proteins independent of the canonical ERAD pathway.

Here we show that EDEM1 is found in dynamic complexes with auto-regulatory function and associates with several canonical ERAD proteins. Deletion of EDEM1 N-terminal IDR impaired its capacity to bind misfolded ERAD substrates and implicitly blocked their EDEM1-induced degradation. We also found that the absence of IDR moderately reduced the association of EDEM1 with some ERAD components whilst enhancing others, supporting the hypothesis that IDR mediates misfolded ERAD client degradation with a higher specificity.

Additionally, we found that EDEM1 overexpression accelerated the degradation of misfolded polypeptides even when proteasomal degradation was severely impaired. We propose this takes place by the generation of protein aggregates and recruitment of the cytosolic autophagy machinery to degrade these structures via ER-phagy receptors, a process coordinated by overexpressed EDEM1 to alleviate ER burden.

## 2. Results

### 2.1. EDEM1 Forms Auto-Regulatory Complexes with ERAD Proteins

We previously found that overexpression of EDEM1 accelerated the degradation of tyrosinase and its mutants via ERAD and was able to bind both calnexin (CNX) and SEL1L [19]. We were interested to understand how EDEM1 cooperates with other ERAD proteins to accelerate misfolded protein degradation. Therefore, we first aimed to identify proteins involved in ERAD that bind or form functional complexes with EDEM1 to efficiently degrade the pool of ER-accumulated proteins amenable to identification by immunoprecipitation and mass-spectrometry analysis.

**Figure 1:**
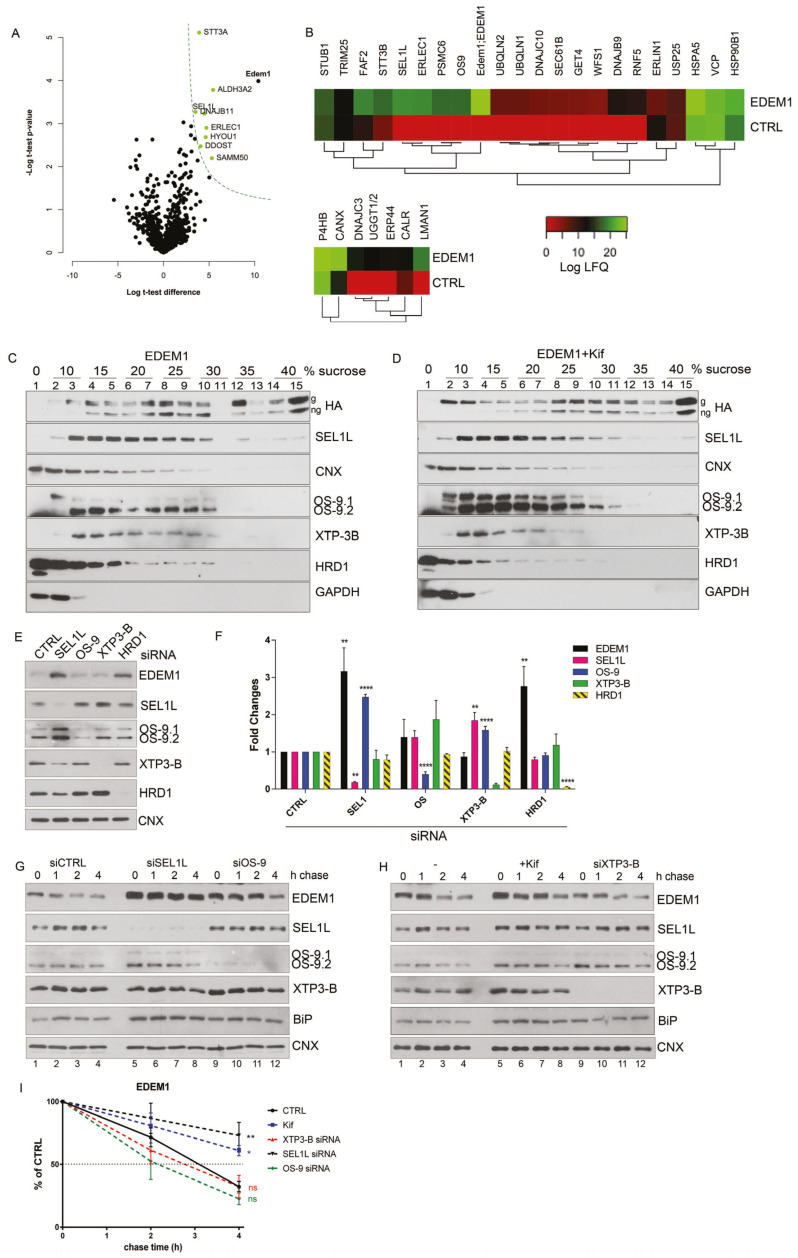
EDEM1 (ER-degradation enhancing α-mannosidase-like) turnover is modulated by ERAD (endoplasmic reticulum-associated protein degradation) proteins. (**A**) Volcano plot of proteins identified using mass spectrometry after enrichment with anti-EDEM1 antibodies from HEK293T cells overexpressing an empty vector (CTRL) or EDEM1 lysed in Triton-X100-containing buffer. Peptides were identified for three biological replicates, assembled in protein groups using MaxQuant, and the label-free quantification (LFQ) values were exported for further analysis. A *t*-test with permutation-based FDR correction was applied to select the statistically significant proteins. Black points represent background proteins, and light green represent significant proteins. EDEM1 is represented in black and bold text. The dash line indicates the threshold for statistically significant proteins (*p* < 0.05 and a minimum log *t*-test difference of 4 was considered). (**B**): All the identified proteins were annotated with Gene Ontologies (GO) biological processes terms from UniProt database, and only entries that contained the ERAD (upper panel) and protein folding (lower panel) key terms were further kept for analysis. The colour key denotes the mean of biological triplicates after the log transformation of the intensity/LFQ values. (**C,D**): HEK293T cells overexpressing EDEM1 treated or not with kifunensine were lysed in a Triton-X100-containing buffer and cleared lysates were subjected to separation on a 10–40% sucrose gradient. Equal volumes of sucrose gradient fractions that were TCA-precipitated and resuspended in 4% SDS buffer, treated (**D**) or not (**C**) with kifunensine were separated in reducing conditions by SDS-PAGE, transferred onto nitrocellulose membranes, and probed with the indicated antibodies: HA (for detecting EDEM1 glycosylated (g) and non-glycosylated (ng) forms), SEL1L, calnexin (CNX), OS-9 (for detecting OS-9.1 and OS-9.2), XTP3-B, HRD1, and GAPDH. (**E**): HEK293T cells were transfected with siRNAs targeting a non-specific sequence (CTRL), alongside siRNA for SEL1L, OS-9, XTP3-B, and HRD1 for 72 h. Cells were harvested, lysed in Triton-X100-containing buffer and processed for SDS-PAGE in denaturing conditions; the proteins were transferred onto nitrocellulose membranes and probed with antibodies for EDEM1, SEL1L, OS-9 (detecting OS9.1 and OS9.2), XTP3-B, HRD1, and CNX as internal control. (**F**): Band densitometry of images present in (**E**) are represented as mean of 3 independent experiments (*n* = 3 ± SEM) and one-way ANOVA comparison with Bonferroni correction was applied for statistical analysis (* *p* < 0.05, ** *p* < 0.01, *** *p* < 0.001, and **** *p* < 0.001). For simplicity of representation, only statistically significant samples are indicated. (**G** and **H**): HEK293T cells were transfected with siRNA targeting a non-specific sequence (CTRL), or siRNA-targeting SEL1L, OS-9, and XTP3-B for 72 h and treated or not (-) with kifunensine (kif) (30 µM/ON); 48 h post transfection cells corresponding to each condition were divided in 4 individual dishes and incubated for another 24 h. Next, the medium was changed with fresh medium supplemented with 50 uM cycloheximide and harvested at the indicated time points. Cells were lysed in Triton-X100-containing buffer, and an equal amount of protein from each sample was prepared for SDS-PAGE in reducing conditions. The levels of endogenously expressed EDEM1, alongside SEL1L, OS-9, XTP3-B, BiP, and calnexin (CNX) were assessed by Western blotting. (**G**): The control (siCTRL), SEL1L (siSEL1L) and OS-9 (siOS-9) siRNA transfected samples. (**H**): Control (-), kifunensine (+kif) and XTP3-B (siXTP3-B) siRNA treated cells. (**I**): Densitometry plot of EDEM1 bands from (**G**) and (**H**), represented as mean of 3 independent experiments (*n* = 3 ± SEM), and one-way ANOVA comparison with Bonferroni correction was applied for statistical analysis (* *p* < 0.05, ** *p* < 0.01, *** *p* < 0.001, and **** *p* < 0.001).

HEK293T cells overexpressing EDEM1 or an empty vector were processed as described in the Materials and Methods section for LC–MS/MS analysis. Three independent experiments were analysed, and a *t*-test with a permutation-based FDR correction was used to delineate co-precipitating proteins. We identified several proteins involved in protein folding, quality control, and ERAD co-precipitating with EDEM1 (Figure 1A). Amongst these, proteins reported to be essential for the degradation of misfolded proteins such as XTP3-B (ERLEC1) [30], SEL1L [31], and DNAJB11 [32], were identified as statistically relevant in our analysis. We further used Gene Ontology (GO) annotation for biological processes to select all identified proteins involved in ERAD, and we performed hierarchical clustering. As shown in the upper panel of Figure 1B, EDEM1 co-clustered with additional members of the ERAD pathway such as OS-9 [30] and PSMC6. An interesting adjacent cluster, with consistent differences between immunoprecipitated EDEM1 and control, contained chaperones involved in protein folding or in disulfide bridge arrangement such as ERdj4 (DNAJB9) and ERdj5 (DNAJC10) [33]. Selecting for proteins involved in glycosylation-dependent folding, we found a sub-cluster including calnexin, the glycosylation folding sensor UDP-Glucose, glycoprotein glucosyltransferase (UGGT1 and UGGT2), a folding oxidoreductase named ERp44 and lectins involved in quality control (calreticulin and ERGIC53) (Figure 1B, lower panel). These results suggest that EDEM1 is found in protein complexes involved in folding and glycosylation, quality control, and protein degradation, thus forwarding the hypothesis that this protein might have multiple functions according to its neighbouring composition.

Next, we analysed the distribution of EDEM1-nucleated complexes by separation on a sucrose gradient in the presence or absence of kifunensine, a chemical compound blocking mannosidase activity that has therefore been proposed to block glycoprotein ERAD. Cell lysates overexpressing EDEM1 were loaded onto a 0–40% sucrose gradient and centrifuged at 39000 rpm for 16h. The proteins corresponding to each fraction were analysed by Western blotting probing with antibodies for EDEM1 and proteins involved in ERAD. As observed in Figure 1C,D, treatment with kifunensine did not dramatically affect the distribution of EDEM1, CNX and GAPDH. However, it induced a mild increase in EDEM1 expression in all fractions. Moreover, treatment with kifunensine caused a shift in the distribution of ERAD proteins (SEL1L, OS-9, XTP-3B, and HRD1) towards lighter complexes and consolidated the expression of OS-9, in particular the OS-9.1 form, suggesting that perturbations of mannosidase-dependent ERAD lead to changes in the solubility of its proteinaceous complexes. 

Previous reports have stated that ERAD complexes are dynamic, and the disruption of complex stoichiometry leads to malfunctioning of protein degradation associated to the ER [34,35,36,37]. Considering we observed differences of expression and distribution for ERAD proteins in the presence of kifunensine, we next aimed to investigate whether the stability of the ERAD complexes including EDEM1 followed this pattern. Thus, we monitored the expression of ERAD proteins when silencing the expression of their partners. As shown in Figure 1E (with quantification in Figure 1F), we observed an increase in EDEM1 and OS-9 levels when SEL1L was silenced by siRNA transfection; moreover, the expression of XTP3-B and HRD1 were slightly decreased under these conditions. Additionally, the knock-down of HRD1 induced an increase in EDEM1 expression and a decrease in SEL1L expression. These results suggest that ERAD complexes are dynamic, and disruption of their stoichiometry leads to an increase or decrease of their counterparts to compensate for this perturbation. 

Furthermore, we performed a cycloheximide chase for cells transfected with siRNA-targeting ERAD components and followed the expression of EDEM1 and other ERAD proteins in parallel with kifunensine treatment. As shown in Figure 1G and H (with a graphical representation in Figure 1I), we observed that the half-life of EDEM1 was substantially increased when silencing SEL1L, as well as in the presence of kifunensine. Additionally, the half-life of EDEM1 was slightly reduced when OS-9 or XTP3-B were knocked-down, thus suggesting the EDEM1-nucleated complexes are dynamic and they compensate for the disruption of stoichiometry by up-regulating the expression of partner proteins. Under these conditions, a mild increase in BiP expression was detected, as shown in Figure 1G and H (fourth panel) for the samples where ERAD components were silenced or after kifunensine treatment, suggesting that the disruption of ERAD components induces mild ER stress and implicitly activates the UPR. However, under the same conditions, calnexin stability was not affected, thus suggesting that the mild activation of the UPR did not cause dramatic changes to the ER glycoprotein metabolism and ER homeostasis.

### 2.2. The ID Region of EDEM1 Is Required for Proteasomal Degradation of ERAD Clients

**Figure 2:**
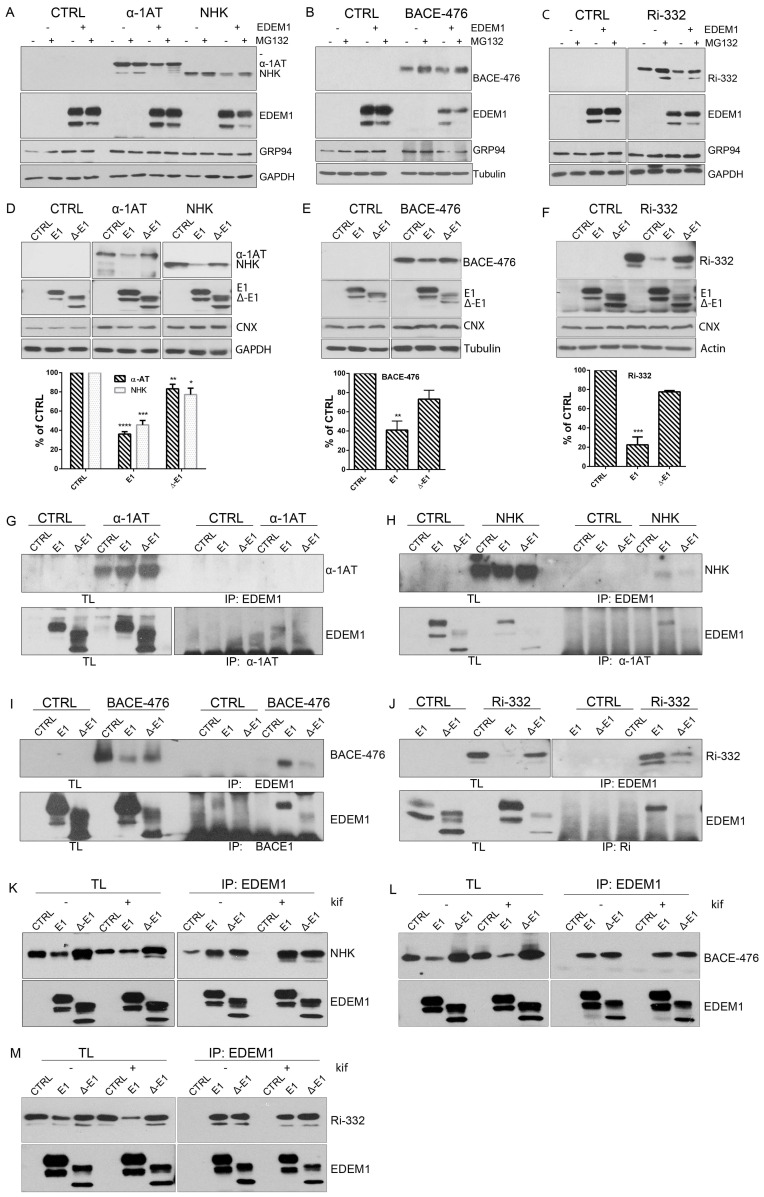
EDEM1 accelerates proteasomal degradation of ERAD substrates. (**A**) HEK293T cells co-expressing an empty vector (CTRL), α -1 antitrypsin (α-1AT), the Null Hong Kong (NHK) mutant with EDEM1, or an empty vector (-) were treated or not with MG132. The cells were lysed in Triton-X100-containing buffer, and an equal amount of protein was separated in polyacrylamide gels in reducing conditions for each sample. Protein expression was detected by Western blotting using antibodies against α-1AT that detected both the α-1AT wild type and NHK mutants, EDEM1, GRP94, or GAPDH. (**B,C**): The same experiment as in (**A**) was performed for BACE-476 (**B**) and Ri-332 (**C**). Proteins expression was detected by Western blotting using antibodies against BACE1 (**B**); ribophorin (**C**); EDEM1, GRP94, and tubulin (**B**); or GAPDH (**C**). (**D**): HEK293T cells co-expressing α-1AT or NHK with an empty vector (CTRL), EDEM1 (E1), or a ∆-EDEM1 (∆-E1) mutant were lysed in Triton-X100-containing buffer. An equal amount of protein from each sample was separated by SDS-PAGE, transferred onto nitrocellulose membrane, and probed with antibodies against α-1AT-detecting wild type form (α-1AT) and NHK mutants, EDEM1-detecting wild type (E1) and truncated (∆-E1) forms, calnexin (CNX), and GAPDH. The band densitometry of four independent experiments is presented in the graph to depict the level of α-1AT and NHK co-expressed with EDEM1 mutants (mean of *n* = 4 ± SEM), and two-way ANOVA comparison with Bonferroni correction was applied for statistical analysis (* *p* < 0.05, ** *p* < 0.01, *** *p* < 0.001, and **** *p* < 0.001). For simplicity only statistically significant samples are indicated. (**E,F**): Same experiment as for (**D**) was performed co-expressing BACE-476 (**E**) and Ri-332 (**F**) with an empty vector (CTRL), EDEM1 (E1) or ∆-EDEM1 (∆-E1) mutant and measured their expression by Western blotting, alongside CNX, tubulin (**E**), and actin (**F**). Band densitometry of 3 independent experiments is represented for each figure (mean *n* = 3 ± SEM), and two-way ANOVA comparison with Bonferroni correction was applied for statistical analysis (* *p* < 0.05, ** *p* < 0.01, *** *p* < 0.001, and **** *p* < 0.001). For simplicity of representation, only statistically significant samples are indicated. (**G**). HEK293T cells transiently transfected to co-express an empty vector (CTRL), EDEM1 (E1), and ∆-EDEM1(∆-E1) with α-1AT were lysed in CHAPS-containing buffer and the cell lysates were subjected to immunoprecipitation with antibodies for EDEM1 (upper panel) or α-1AT (lower panel). The eluted samples were separated by SDS-PAGE and probed with antibodies for the co-precipitated proteins. (**H**–**J**): Same as in (**G**) for NHK, BACE-476 and Ri-332, respectively, the eluted samples were separated by SDS-PAGE and probed with antibodies for the co-precipitated proteins α-1AT (H), BACE1 (**I**), Ribophorin I (**J**) (upper panels) and EDEM1 (lower panels). (**K**): HEK293T cells knock-out for EDEM1 were co-transfected with an empty vector (CTRL), EDEM1 (E1), ∆-EDEM1(∆-E1) and NHK, as in G and 24h after transfection kifunensine (kif) was added to half of the samples. Following, they were lysed in CHAPS-containing buffer, processed for immunoprecipitation with antibodies for EDEM1, and used for Western blotting with antibodies against α-1AT (upper panel) and EDEM1 (lower panel). (**L**) and (**M**) Same experiment as in (**K**) was performed for BACE-476 and Ri-332, respectively.

**Figure A1:**
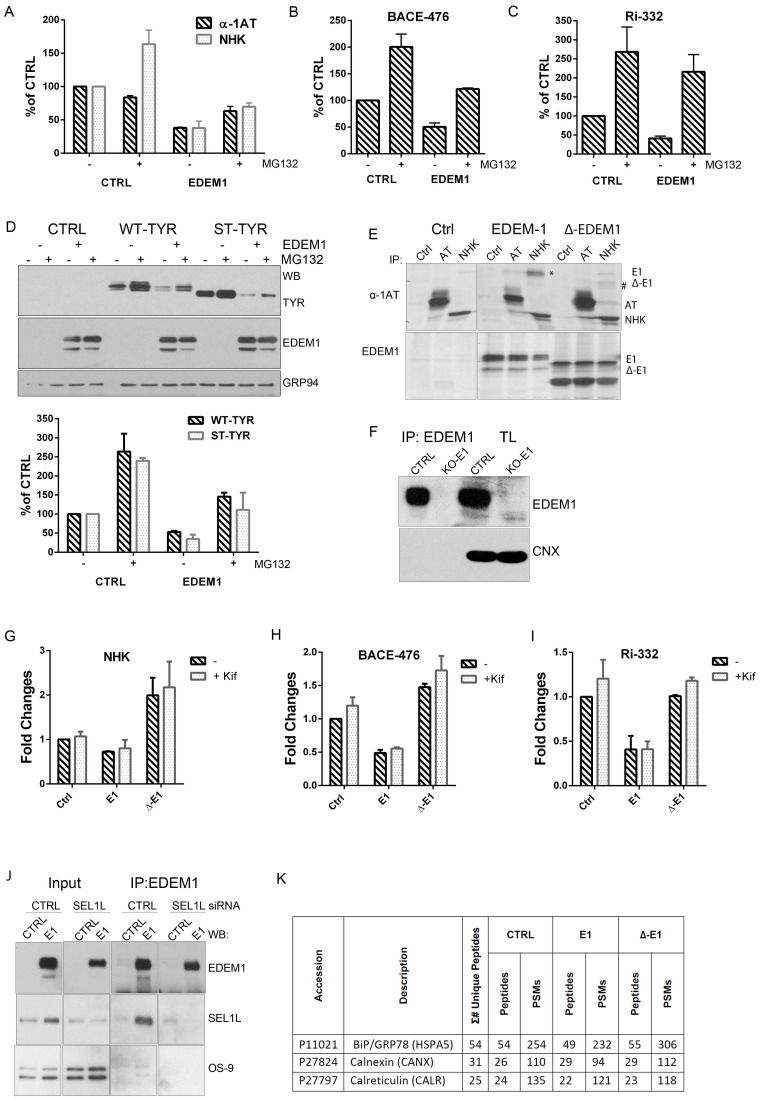
**(A**–**C)** Band densitometry plots of α-1AT and NHK (**A**), BACE-476 (**B**), and Ri-332 (**C**), co-expressed in HEK293T with an empty vector (CTRL) or EDEM1 (EDEM1), treated or not with MG132, as presented in Figure 2A–C (mean *n* = 2 ± SEM). (**D**) Cells overexpressing EDEM1 alongside an empty vector (CTRL), WT-Tyr, or its truncated variant ST-Tyr were treated or not with MG132. The pelleted cells were lysed in a Triton-X100-containing buffer, and equal amounts of proteins were separated in reducing conditions by SDS-PAGE. Protein expression was detected by Western blotting using antibodies against α-Tyr, EDEM1, or GRP94 (upper panel); the lower panel depicts band densitometry plots for WT-Tyr and ST-Tyr (mean *n* = 2 ± SEM). (**E**) HEK293T co-expressing an empty vector (CTRL), EDEM1 (E1), or Δ-EDEM1 (Δ-E1) with α-AT (AT) and NHK were metabolically labelled with ^35^S-Met/Cys for 20 min. Harvested cells were lysed in a CHAPS-containing buffer, and cleared lysates were subjected to immunoprecipitation with antibodies for α-AT (upper panel) and EDEM1 (lower panel). The eluted complexes were separated by SDS-PAGE followed by autoradiography. (**F**) HEK293T (CTRL) and HEK293T-KO for EDEM1 (KO-E1) cells were lysed in a TritonX-100-containing buffer, and cleared lysates were immunoprecipitated with EDEM1 antibodies, captured on Protein A-Sepharose and eluted with a 1× Laemmli buffer. The elutions corresponding to each condition were separated by SDS-PAGE, transferred onto nitrocellulose membrane, and probed for EDEM1 and CNX. (**G-I**) The band densitometry plots of bands detected for total lysates (TL) of NHK (**G**), BACE-476 (**H**), and Ri-332 (**I**), co-expressed in HEK293T-KO with an empty vector (CTRL), EDEM1 (EDEM1), or ∆-EDEM1 (∆-E1) treated or not with kifunensine (kif), as presented in Figure 2K–M (mean *n* = 2 ± SEM). (**J**) HEK293T cells were transfected with siRNA for a non-coding RNA sequence (CTRL) and SEL1L for 72 h. In the last 24 h of transfection, the cells were transfected with a plasmid encoding for EDEM1 (E1) or an empty vector (CTRL). The samples lysed in a CHAPS-containing buffer were immunoprecipitated with antibodies against EDEM1, captured on Protein A-Sepharose, and eluted with a 1× Laemmli buffer; the eluted samples were used for Western blotting, and the membranes were probed with antibodies against EDEM1, SEL1L, and OS-9. (**K**) Expression of BiP, CNX, and calreticulin for total lysates of HEK293T cells expressing an empty vector, wild type EDEM1, or Δ-EDEM1 analysed by mass spectrometry-based proteomics. The total number of peptides and PSMs identified for each protein is indicated in the figure.

We have previously shown that the IDR of EDEM1 located at its N-terminus is required for accelerating the degradation of tyrosinase and its mutants [19]; therefore, we hypothesised that IDR-deficient EDEM1 would also impair the degradation of other reported ERAD clients. To test this hypothesis, we used previously characterized glycosylated ERAD substrates: α1-antitrypsin (α-1AT), Null Hong Kong (NHK), Ribophorin (Ri)-332, and beta-secretase (BACE)-476 [38,39,40]. We first confirmed the EDEM1 dependency for the proteasomal degradation of these substrates and found that the inhibition of proteasome activity almost entirely rescued the EDEM1-induced degradation of the ERAD clients (Figure 2A–C and Figure A1A–D).

First, we analysed the expression of BiP, CNX, and calreticulin for the total lysates of HEK293T cells expressing an empty vector, wild type EDEM1, or Δ-EDEM1 using mass spectrometry-based proteomics to obtain a relative quantitative assessment of protein expression. As shown in Figure A1K, we found no notable differences between the three cell lines in terms of BiP, CNX, and calreticulin expression based on the number of peptides and peptide spectrum matches (PSMs) identified after whole proteome analysis, so we can exclude a major activation of the UPR upon transfection of both EDEM1 mutants.

Next, we co-expressed EDEM1 and the EDEM1 lacking its IDR (∆-EDEM1) alongside ERAD clients and monitored their expression by Western blotting. As shown in Figure 2D, the degradation of α-1AT and NHK was considerably enhanced in the presence of EDEM1, but not in that of ∆-EDEM1. Similarly, the degradation of BACE-476 and Ri-332 was partially impaired when co-expressed with ∆-EDEM1 (Figure 2E and F, respectively). Altogether, these results confirmed our hypothesis that the IDR of EDEM1 is required for accelerating the degradation of other ERAD clients than tyrosinase and its mutants. 

As we previously showed that the IDR was also required for an efficient interaction with tyrosinase mutants, we tested the interaction of EDEM1 mutants with the client proteins by immunoprecipitation and Western blotting [19]. We found that ∆-EDEM1 bound with less affinity the client proteins for all tested model ERAD substrates (Figure 2G–J). We also confirmed the interaction of EDEM1 mutants with α-1AT and NHK by pulse-chase and immunoprecipitation, a more sensitive method to assess protein–protein interactions. As shown in Figure A1E, we found that EDEM1 was efficiently co-precipitated with both α-1AT and NHK, whereas the band corresponding to ∆-EDEM1 was not as prominent for NHK or practically undetectable for α-1AT.

Although the overexpression of a mutant form of a protein usually has a dominant effect and the observed behaviour is mostly accounted to the mutant form, to fully confirm the role of EDEM1 IDR in substrate binding and degradation, we used an EDEM1-deficient cell line. We confirmed the requirement of IDR for accelerating the degradation and interaction with ERAD substrates by co-expression with EDEM1 and ∆-EDEM1 in HEK293T cell knock-out for EDEM1 in the presence or absence of kifunensine. First, we verified the absence of EDEM1 in the knock-out (KO) cell line by immunoprecipitation with EDEM1 antibodies and probed for EDEM1 and calnexin; we could not detect the expression of EDEM1 in the E1-KO cells compared to the parental cell line (Figure A1F). As shown in Figure 2K (with quantification Figure A1G), the degradation of NHK was accelerated in the presence of EDEM1 but not ∆-EDEM1, and the latter bound with less affinity the substrate when compared with the total available amount of substrate. Similar results were obtained when co-expressing EDEM1 mutants with BACE-476 and Ri-332 as shown in Figure 2L and M (with quantification in Figure A1H and A1I, respectively). A mild accumulation of the ERAD substrates could be detected by treatment with kifunensine, although not to the expected level, which might be explained by the fact that deletion of a mannosidase protein, in this case EDEM1, desensitized the cells to kifunensine treatment, likely requiring a higher concentration of drug to be effective. Additionally, the presence of kifunensine did not interfere with interaction of EDEM1 or ∆-EDEM1 with ERAD substrates (Figure 2K–M). In conclusion, our results suggest that deletion of EDEM1 IDR reduced its binding affinity for ERAD clients and impaired the EDEM1-induced degradation of all the tested proteins.

### 2.3. EDEM1 and ∆-EDEM1 Bind with Different Affinities ERAD Proteins and Form Complexes of Variable Solubility

**Figure 3:**
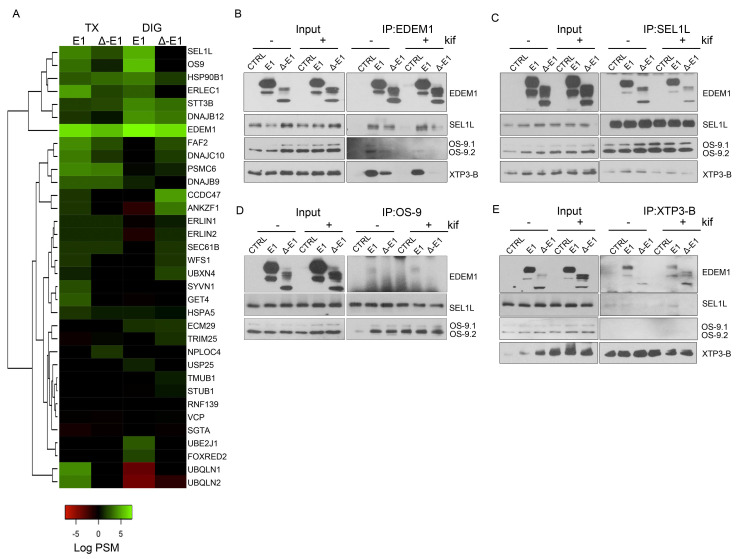
Association of EDEM1 and ∆-EDEM1 with ERAD partner proteins. (**A**). Heatmap representation of proteins identified by mass spectrometry after enrichment with anti-EDEM1 antibodies from HEK293T cells overexpressing EDEM1 or ∆-EDEM1 extracted in Triton X-100 (TX) or digitonin (DIG)-containing buffers. The identified proteins were annotated with GO terms from UniProt database, and only entries that contained the ERAD key-term were further kept for analysis. The colour key denotes spectral counts (SC) in log scale after negative control (cells transfected with an empty vector and immunoprecipitated with EDEM1 antibodies) subtraction. (**B**–**E**). HEK293T cells were transfected to overexpress an empty vector (CTRL), EDEM1 (E1), and ∆-EDEM1 (∆-E1), and they were treated or not with 30 µM kifunensine (kif) ON. Cells were harvested, lysed in CHAPS-containing buffer, and equal amounts of protein were used for immunoprecipitation with antibodies for: (**B**) EDEM1, (**C**) SEL1L, (**D**) OS-9, and (**E**) XTP3-B. The eluted resin-bound complexes were separated by SDS-PAGE, and the membranes were probed with antibodies for EDEM1, SEL1L, OS-9, and XTP3-B.

To test whether the binding of EDEM1 to ERAD proteins was modified by deletion of its intrinsically disordered region, we monitored the proteins co-precipitated with EDEM1 by lysing cells overexpressing EDEM1 or ∆-EDEM1 in buffers containing two different detergents: Triton-X100 and Digitonin. These detergents have different capacities of extracting proteins and maintaining protein complexes, as described previously [10,12]. Equal amounts of total protein were used for immunoprecipitation with rabbit anti-EDEM1 antibodies, as previously described [19]; the eluted samples were loaded onto SDS-PAGE gels, and separated proteins were in-gel digested with sequencing grade trypsin. Extracted peptides were analysed by LC–MS/MS. As shown in Figure 3A. EDEM1 co-precipitated when using both Digitonin and Triton X-100, with a cluster of canonical ERAD members (SEL1L, OS-9, ERLEC1/XTP3-B, and GRP94/HSP90B1 and DNAJB12); however, most of these associations were reduced for Δ-EDEM1. Moreover, our experiments also allowed us to assess the strength of association for proteins co-precipitated with EDEM mutants, which are sensitive to the extraction conditions.

Overall, we found that EDEM1 bound with higher affinity proteins involved in ERAD compared to ∆-EDEM1, and the clusters of proteins identified co-precipitating with EDEM1 mutants were variable with the strength of the detergent. This suggests that EDEM1, when lacking its IDR, binds less efficiently to some of the canonical ERAD proteins, regardless of the extraction method; however, Δ-EDEM1 bound, with higher affinity than EDEM1, a cluster of proteins described as involved in ERAD, different form the canonical described proteins (ERLIN1, ERLIN2, ECM29, TRIM25, etc). One possibility is that ∆-EDEM1 associates with these proteins to increase its stability; as we have previously shown, the protein is less stable than its wild-type counterpart [19] or for its own degradation, a process reported for several proteins involved in ERAD and the UPR [31,41,42].

Further, we aimed to validate some of the binding partners identified by mass spectrometry analysis through immunoprecipitation and Western blotting experiments. HEK293T cells were transfected to overexpress EDEM1 or ∆-EDEM1 in the presence of absence of kifunensine, and then they were lysed and used for immunoprecipitation with antibodies against EDEM1, SEL1L, OS-9, or XTP3-B. Eluates were loaded onto SDS-PAGE gels, transferred onto nitrocellulose membranes, and probed with antibodies for EDEM1, SEL1L, OS-9, and XTP3-B. As shown in Figure 3B–E, EDEM1 bound the canonical ERAD proteins even in the presence of kifunensine, although this binding was weaker, suggesting that the formed complexes were sensitive to disruption of N-glycan processing and protein homeostasis dysregulation, as previously reported by us for EDEM3 [13]. In particular, the association of ∆-EDEM1 with XTP3-B was more prominent for the non-glycosylated form in the absence of kifunensine, and a consolidation of the glycosylation form was observed in the presence of kifunensine.

We also tested the binding of EDEM1 with OS-9 in the presence or absence of SEL1L because it has been previously suggested that they are forming a functional complex [34]; therefore, cells were transfected with siRNA for SEL1L and plasmid to overexpress EDEM1. The cells were lysed, used for immunoprecipitation with antibodies against EDEM1, and probed for EDEM1, SEL1L, and OS-9. We found that the association of EDEM1 with OS-9 was not detected for the samples transfected with siRNA for SEL1L, thus confirming the hypothesis of a functional complex (Figure A1J).

### 2.4. EDEM1 and Not ∆-EDEM1 Induces ERAD Clients Degradation When ERAD Complexes Are Disrupted

The E3 ubiquitin-ligase HRD1, along with its adaptor protein SEL1L, was proposed to be the central player in protein dislocation from the ER, and the degradation of several substrates was found to be HRD1-dependent [43,44,45]. Additionally, OS-9 and XTP3-B were shown to be key factors in ERAD, and, as shown by our previous results, all these proteins were found to be co-precipitated with EDEM1. We hypothesised that if EDEM1 was assisted by any of these ERAD components, when silenced, the EDEM1-induced degradation of the misfolded substrates would be blocked. Moreover, in the same experiments, we tested the capacity of ∆-EDEM1 to accelerate client degradation when ERAD components were silenced.

**Figure 4:**
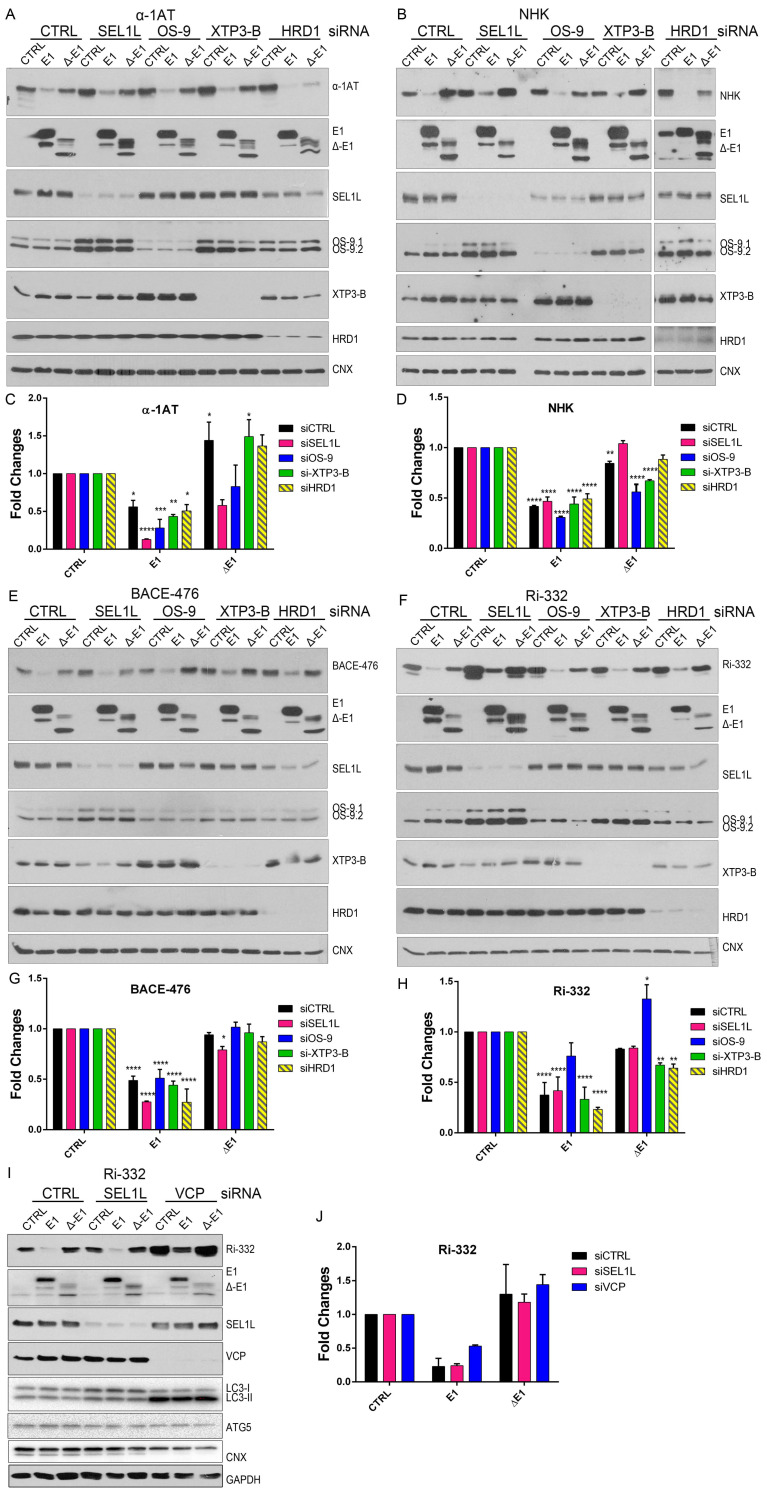
EDEM1 accelerates the degradation of misfolded polypeptides even when ERAD is not functional. (**A**) HEK293T cells were transfected with siRNA targeting a non-specific sequence (CTRL), SEL1L, OS-9, XTP3-B, and HDR1 for 72 h; during the last 24 h, another transfection was made to overexpress an empty vector (CTRL), EDEM1 (E1), and ∆-EDEM1 (∆-E1), along with α-1AT. Cells were harvested, lysed in Triton-X 100-containing buffer, and an equal amount of protein from each sample was separated by SDS-PAGE in reducing conditions. Protein expression was estimated by Western blotting using antibodies against α-1AT, EDEM1, SEL1L, OS-9, XTP3-B, HRD1, and CNX as an internal control. (**B**): The same experiment was performed for NHK and its expression, and the above-mentioned proteins were detected by Western blotting. (**C**,**D**) Band densitometry plot of α-1AT and NHK presented in (**A**) and (**B**), respectively (mean *n* = 3 ± SEM), and two-way ANOVA comparison with Bonferroni correction was applied for statistical analysis (* *p* < 0.05, ** *p* < 0.01, *** *p* < 0.001, and **** *p* < 0.001). For simplicity only statistically significant samples are indicated. (**E,F**) During the last 24 h of siRNA transfection, BACE-476 and Ri-332 were co-expressed with an empty vector (CTRL), EDEM1 (E1), and ∆-EDEM1 (∆-E1) in HEK293T, in a similar experiment to that in (**A**). The protein expression was estimated by Western blotting using antibodies against BACE1 (**E**) and Ribophorin I (**F**) alongside EDEM1, SEL1L, OS-9, XTP3-B, HRD1, and CNX as an internal control. (**G,H**) Band densitometry plot of BACE-476 and Ri-332 presented in (**E**) and (**F**), respectively (mean *n* = 3 ± SEM), and two-way ANOVA comparison with Bonferroni correction was applied for statistical analysis (*p < 0.05, **p < 0.01, ***p < 0.001, and ****p < 0.001). For simplicity of representation, only statistically significant samples are indicated. (**I**): HEK293T cells were transfected with siRNA that targeted a non-specific sequence (CTRL), SEL1L, or p97/VCP for 72 h; during the last 24 h, the cells were transfected to co-express Ri-332 alongside an empty vector (CTRL), EDEM1(E1), and ∆-EDEM1 (∆-E1). Samples were processed as in (**A**), and the membranes were probed with antibodies for Ribophorin-I, EDEM1, SEL1L, VCP, LC3, ATG5, CNX, and GAPDH. (**J**): Band densitometry plot of Ri-332 is presented in (**I**) (mean *n* = 2 ± SEM).

In this regard, we transfected cells with siRNA-targeting SEL1L, HRD1, OS-9, or XTP3-B, and we monitored the effect that EDEM1 and ∆-EDEM1 had over ERAD substrates. HEK293T cells were transfected with siRNAs for SEL1L, HRD1, OS-9, or XTP3-B for 72 h, and throughout the last 24 h, cells were co-transfected with EDEM1 mutants and ERAD substrates α-1AT, NHK, BACE-476, and Ri-332, respectively. Cells were harvested and lysed, and equal amounts of protein were loaded to SDS-PAGE for each sample. As observed in Figure 4A and quantification 4C, an accumulation of α-1AT in the absence of SEL1L and HRD1 was obvious for empty-vector transfected samples, suggesting that the degradation of α-1AT is mediated by SEL1L and/or HRD1. Conversely, the degradation of α-1AT was accelerated in the presence of EDEM1 in all samples, including the siRNA-transfected samples. As expected, ∆-EDEM1 did not affect the degradation of α-1AT to the same extent as EDEM1 under these conditions, confirming the hypothesis that the IDR is required for the accelerated degradation of ERAD proteins induced by EDEM1. NHK was used for an experiment in the same conditions as above, and we observed for an accelerated degradation samples co-transfected with EDEM1, similar to α-1AT (Figure 4B; see quantification in Figure 4D).

**Figure A2:**
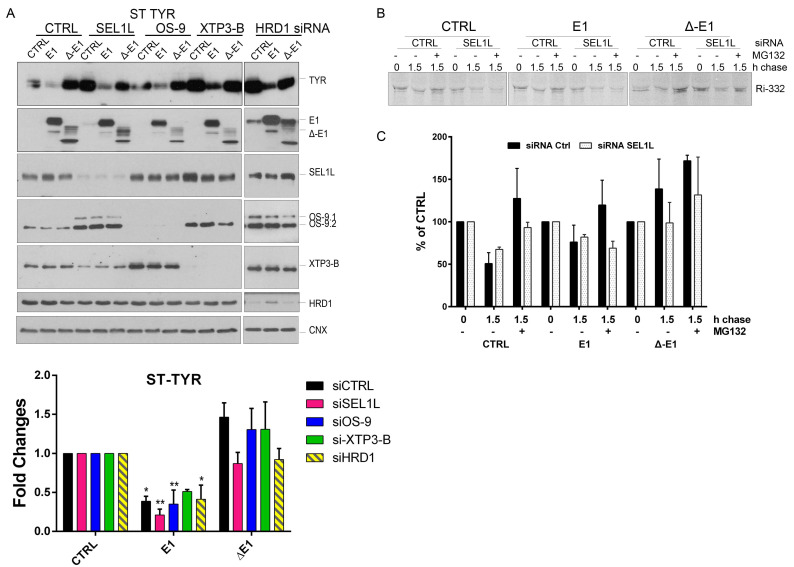
(**A**) HEK293T cells were transfected with siRNA targeting a non-specific sequence (CTRL), SEL1L, OS-9, XTP3-B and HRD1 for 72 h; during the last 24 h another transfection was made to overexpress an empty vector (CTRL), EDEM1 (E1), and mutant ∆-EDEM1 (∆-E1) along with ST-Tyr. Cells were harvested and lysed, and the expression of Tyr, EDEM1, SEL1L, OS-9, XTP3-B, HRD1, and CNX as an internal control was estimated by Western blotting (upper panel); the lower panel depicts band densitometry plots of ST-Tyr (mean *n* = 3 ± SEM), and a two-way ANOVA comparison with a Bonferroni correction was applied for statistical analysis (* *p* < 0.05, ** *p* < 0.01, *** *p* < 0.001, and **** *p* < 0.001). For simplicity of representation, only statistically significant samples are indicated. (**B,C**) HEK293T were transfected with siRNA-targeting SEL1L or a non-specific sequence (CTRL) for 72 h; for the last 24 h, the cells were transiently transfected to co-express an empty vector (CTRL), EDEM1 (E1), or Δ-EDEM1 (Δ-E1) along with Ri-332. Cells treated or not with MG132 were metabolically labelled with ^35^S-Met/Cys for 20 min and chased for the indicated time points. Cells were harvested lysed in a Tritonx-100-containing buffer, and cleared lysates were subjected to immunoprecipitation with antibodies for Ribophorin I. The eluted samples were separated by SDS-PAGE followed by autoradiography. The band intensity of 2 independent experiments was estimated by densitometry measurement (using ImageJ software) as represented in the lower panel of the figure (mean *n* = 2 ± SEM).

The same experiment was performed for BACE-476 (Figure 4E; quantification in Figure 4G) and Ri-332 (Figure 4F; quantification in Figure 4H); co-expression with EDEM1 led to an accelerated degradation that was independent of the level of endogenous SEL1L, OS-9, XTP3-B, and HRD1. Similar results were obtained for the previously reported ERAD substrate ST-Tyr, which had an accelerated degradation in the presence of EDEM1 but not ∆-EDEM1, see the upper panel of Figure A2A, with quantification in the lower panel. Overall, these results suggest that EDEM1 overexpression can bypass most of the proteins that have been proposed to function as canonical ERAD to efficiently degrade proteins accumulated in the ER, and in the absence of its IDR, EDEM1 is unable to retain this capacity.

Next, we questioned whether EDEM1 overexpression accelerated the proteasomal degradation of the substrates by an alternative dislocation pathway or it might involve the autophagy machinery as back-up. In this regard, we performed a similar experiment to the one presented in Figure 4F and monitored the expression of Ri-332 when SEL1L or p97/VCP, an AAA-ATPase with critical function in proteasomal degradation, were silenced. Surprisingly, we found that overexpression of EDEM1, and not ∆-EDEM1, could accelerate Ri-332 degradation in the absence of p97/VCP. This prompted us to check the activation of autophagy by monitoring the lipidation of LC3 and the expression of ATG5, two essential proteins for the autophagy process. We observed a moderate LC3 lipidation for the samples where SEL1L was silenced and a considerable increase for the samples where p97/VCP was silenced, suggesting an enhanced activation of autophagy under these conditions (Figure 4I; see quantification in Figure 4J). The degradation pattern of Ri-332 was also confirmed by pulse-chase and immunoprecipitation, as shown in Figure A2B (see quantification in Figure A2C), supporting the hypothesis that EDEM1 overexpression accelerates ERAD clients degradation even when proteasomal degradation is impaired.

### 2.5. EDEM1 Overexpression Induces the Formation of Oligomers for ERAD Substrates Facilitating Their Degradation via Autophagy 

**Figure 5:**
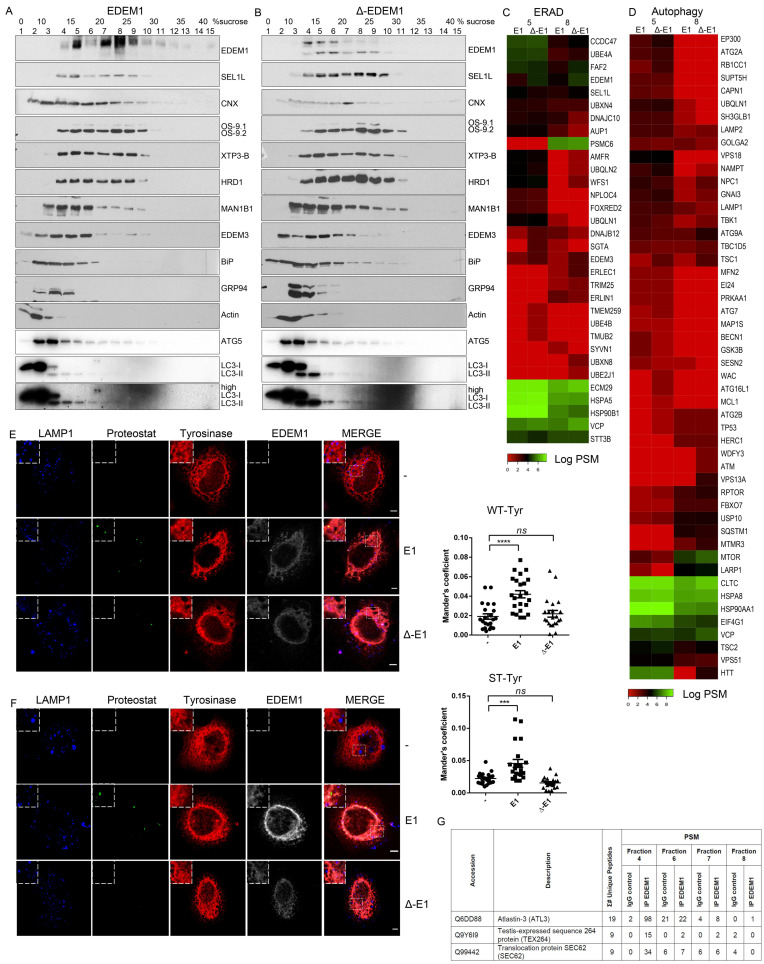
EDEM1 induces the formation of aggregates and targets them for degradation by ER-phagy. (**A,B**) Cells overexpressing EDEM1 and ∆-EDEM1, respectively, were lysed in Digitonin-containing buffer, and cleared lysates were subjected to separation on a 10–40% sucrose gradient. Equal volumes of sucrose gradient fractions that were TCA-precipitated and resuspended in 4% SDS buffer were separated in reducing conditions by SDS-PAGE, transferred onto nitrocellulose membranes, and probed with antibodies against the indicated proteins: EDEM1, SEL1L, calnexin (CNX), OS-9, XTP3-B, HRD1, MAN1B1, EDEM3, BiP, GRP94, Actin, ATG5, and low exposure (LC3-I and II) and high exposure (high LC3-I and II) LC3. Heatmaps of ERAD (**C**) and autophagy (**D**) identified proteins using mass spectrometry in two sucrose gradient fractions, chosen based on EDEM1 expression as identified in (**A**) (2 maximum expression peaks that suggest 2 different complexes). TCA-precipitated proteins corresponding to each selected fraction (as shown by Western blotting), were separated by SDS-PAGE, followed by in-gel digestion protocol described in the Materials and Methods section, and subjected to LC–MS/MS analysis. Identified proteins were annotated with GO terms from UniProt and entries that contained the ERAD (**C**) and autophagy (**D**) key-terms were further kept for analysis. Heatmap of spectral counts distribution in log scale for the selected proteins is presented. (**E**) HeLa cells were co-transfected with WT-tyrosinase (WT-Tyr) and an empty vector (-), EDEM1 (E1), or ∆-EDEM1 (∆-E1); 24 h post-transfection, cells were fixed with 1% PFA for 1h and processed as described in the Materials and Methods section. Confocal images for LAMP1, Proteostat, tyrosinase, and EDEM1 are presented; the scale bar represents 5 µM. The co-localization between tyrosinase and Proteostat was evaluated by calculating Mander’s correlation coefficient using the JACoP plugin (mean *n* = 20 ± SEM). Two-way ANOVA comparison with Bonferroni correction was applied for statistical analysis (* *p* < 0.05, ** *p* < 0.01, *** *p* < 0.001, and **** *p* < 0.001; ns—non-significant). Insets of 10.5 μM (zoom image on the indicated area) are inserted in each picture for a higher magnification. (**F**): The same experiment as in (**E**) was performed for the soluble form of tyrosinase (ST-Tyr) and the co-localization of ST with Proteostat was evaluated, a mean of *n* = 19 ± SEM is represented graphically, and two-way ANOVA comparison with Bonferroni correction was applied for statistical analysis (* *p* < 0.05, ** *p* < 0.01, *** *p* < 0.001, and **** *p* < 0.001; ns—non significant). Insets of 10.5 μM (zoom image on the indicated area) are added to each image for a higher magnification. (**G**): This depicts peptide spectrum matches (PSMs) and the total number of unique peptides identified for three of the six reported ER-phagy receptors that were identified after immunoprecipitation with EDEM1 and the LC-MS/MS detections of sucrose gradient fractions, processed as described in the Materials and Methods section.

The above-mentioned results suggest EDEM1 might accelerate the degradation of misfolded proteins accumulated in the ER by shifting the system towards autophagy; to test this hypothesis, we first evaluated how EDEM1 and ∆-EDEM1 co-fractionated with proteins of the ERAD pathway, that had already been validated in our previous experiments, along with LC3 and ATG5 (proteins involved in autophagy). We observed EDEM1 co-fractionates with several proteins involved in folding such as calnexin, BiP, and GRP94, with ERAD proteins like SEL1L, OS-9, XTP3-B, EDEM3, MAN1B1, and HRD1, as well as ATG5 and LC3. As shown in Figure 5A,B, ATG5 and LC3 were concentrated towards lighter fractions, partially overlapping with EDEM1 and ∆-EDEM1, with a more prominent concentration of LC3 for ∆-EDEM1. 

Next, we assessed the co-fractionation of EDEM1 and ∆-EDEM1 by spectral counts abundance in the LC–MS/MS analysis for the fractions with the highest abundance for EDEM1 presented in Figure 5A. We annotated all identified proteins using the UniProt GO service and selected only entries associated with key-terms for ERAD and autophagy. For autophagy, we increased the stringency of the analysis by only selecting proteins included in the Human Autophagy Modulator Database (HAMDB), a resource containing proteins, chemicals, and microRNAs related to the autophagy pathway [46]. As expected, we found several proteins involved in ERAD co-fractionating with EDEM1 mutants with small differences between EDEM1 and ∆-EDEM1 (Figure 5C). Assessing the distribution of autophagy-related proteins, we found several core-components of the autophagy machinery, such as ATG2A, ATG2B, ATG9A, ATG7, ATG16L1, Beclin1, and p62/SQSTM1 co-fractionating with EDEM1 mutants, as shown in Figure 5D.

**Figure A3:**
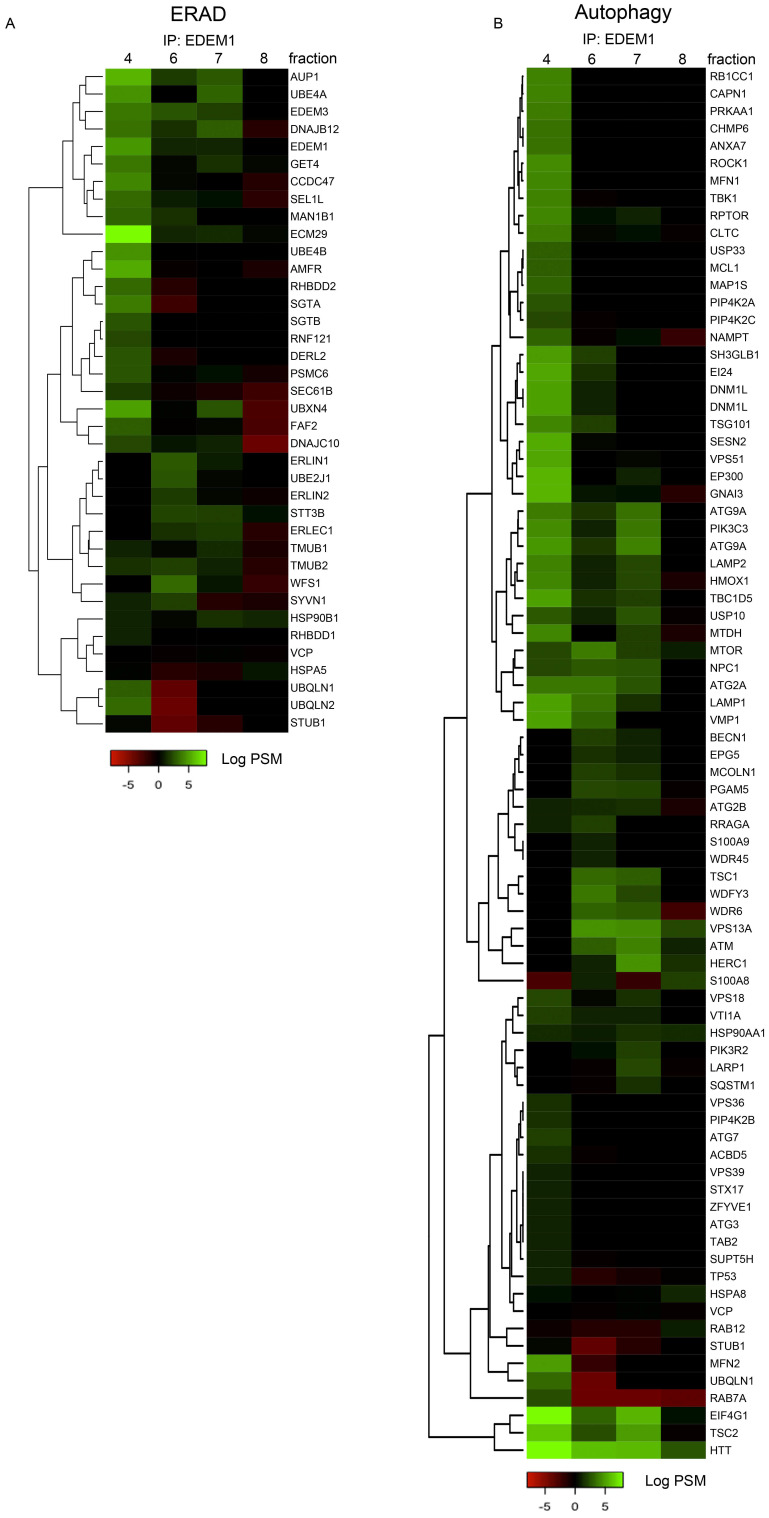
Heatmap of ERAD (**A)** and autophagy (**B**) proteins identified, using mass spectrometry for the indicated sucrose gradient fractions that were immunoprecipitated with EDEM1; the eluted samples were separated by SDS-PAGE, followed by in the gel digestion protocol, as described in the Materials and Methods section. Identified proteins were annotated with GO terms from UniProt database, and entries containing the ERAD (**A**) and autophagy (**B**) key-terms were further analysed. Positive values, after negative control subtraction of spectral counts in log scale corresponding to each extraction condition, are represented as a heatmap.

Moreover, fractions 4, 6, 7, and 8 of cells overexpressing EDEM1 were used for immunoprecipitation with EDEM1 antibodies, and the eluted samples were separated by SDS-PAGE and processed for mass-spectrometry analysis, as described in Materials and Methods. Proteins annotated with ERAD as keyword in the UniProt database are represented in the heatmap in Figure A3A. Similarly, proteins annotated as involved in autophagy, in both UniProt GO annotation and the HAMDB, are represented in Figure A3B. We identified several components of the core machinery for autophagy co-precipitating with EDEM1 (such as ATG2A, ATG2B, ATG3, ATG7, ATG9A, Beclin1, VMP1, and p62/SQSTM1) with different abundance according to the analysed fraction, thus supporting our initial hypothesis that EDEM1 might be able to interact with the autophagy machinery. However, since most of the autophagy core machinery is composed of cytoplasmic proteins, it is unlikely that these components would bind directly to EDEM1; therefore, we hypothesised this task could be mediated by one or more of the ER-phagy receptors previously reported in the literature [47,48,49,50,51,52]. Out of the six ER-phagy receptors reported up to date, we identified three that co-precipitated with EDEM1 in the upper-mentioned fractions (Figure 5G), thus confirming that EDEM1 could bind the autophagy machinery via an ER-phagy receptor, potentially with the purpose to accelerate the degradation of ER accumulated proteins when ERAD is impaired. 

**Figure A4:**
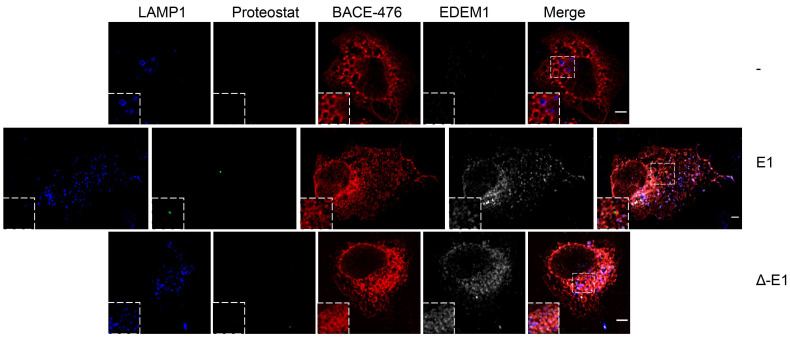
The same experiment as in Figure 5E were performed, and BACE-476, along with an empty vector (-), EDEM1 (E1), or mutant ∆-EDEM1(∆-E1), was co-transfected. The images presented were acquired with confocal microscope and the scale bar on the main image is 5 μM. Zoom image is 10.5 μM.

Recent reports have stated that IDRs mediate the formation of biomolecular condensates that can change the biological function of molecules such as proteins or RNA [53,54,55]; additionally, EDEM1 has previously been reported to be involved in disposal of orphan oligomers from the ER [21,22,24]. We therefore hypothesised that the IDR of EDEM1 could act as driver for the formation of these structures when ER is overloaded, and it could potentially shift their degradation for autophagy. For this, HeLa cells seeded on coverglasses were transfected to co-express an empty vector or EDEM1 mutants alongside either WT or ST-tyrosinase, and these were processed for immunofluorescence, as described in the Materials and Methods section. We evaluated the formation of amyloid-like oligomeric structures by staining with Proteostat, a dye with specificity for β-sheet-rich structures. As shown in Figure 5E (WT-Tyr) and 5F (ST-Tyr), the overexpression of EDEM1 induced an increase in the co-localisation of overexpressed tyrosinase with Proteostat, while ∆-EDEM1 did not, as shown by the graphically represented Mander’s coefficient values. Furthermore, when BACE-476 was used for the same experiment, similar results were obtained (Figure A4). Overall, our results suggest that EDEM1 drives the formation of the oligomers of misfolded proteins, while ∆-EDEM1 does not; this process is likely driven by the IDR of EDEM1. This could explain why the overexpression of EDEM1 still accelerated misfolded protein degradation even in the absence of a functional ERAD or proteasome, and we speculate that EDEM1 can achieve this task by driving the formation of aggregates of misfolded proteins and recruiting the autophagy machinery via interaction with ER-phagy receptors.

## 3. Discussion

Protein quality control in the endoplasmic reticulum is a tightly regulated process, and any dysregulation of this process can lead to pathological conditions. In particular, the process of recognition and targeting for the degradation of misfolded proteins is not completely elucidated, despite the sustained efforts of many labs in the last few years. ERAD has been described as a tightly regulated and dynamic process that ensures the endoplasmic reticulum disposes the excess folding-incompetent proteins to overcome proteomic burden. EDEM proteins have been described as key players for ERAD by acting as mediators between the folding and degradation machinery, prevalently recognising glycoproteins. EDEM1 was initially proposed to extract glycoproteins from the calnexin folding cycle and target them for proteasomal degradation by association with ERAD components to facilitate their dislocation to the cytosol [4,5]. However, our lab and others have shown that EDEM1 is able to bind and accelerate the degradation of non-glycosylated proteins or in conditions where glycan recognition is blocked [15,17,19]. This has been attributed to the presence of an intrinsically disordered region at its N-terminus, which mediates the association with misfolded proteins based on their capacity to expose hydrophobic patches [19,56].

We documented using mass spectrometry, that the proteins co-enriched with EDEM1 are involved in folding and glycosylation, quality control, and protein degradation, suggesting EDEM1 could form different complexes, thus having versatile functions. It is worth mentioning that using LC–MS/MS analysis, we found a relatively reduced number of interactors involved in the ER folding system, such as calnexin, glucosyltransferase (UGGT1 and UGGT2), folding enzymes (Erp44), and the lectins calreticulin and ERGIC-53. In exchange, a significantly higher number of ER resident proteins associated with the ERAD pathway were detected as EDEM1-associated proteins. Our experiments suggest that the stability endogenously expressed EDEM1 is modulated by the abundance and complex stoichiometry of proteins shown to be involved in ERAD substrate degradation. Knock-down experiments indicated that the level of endogenously expressed EDEM1 is modulated by the OS-9-SEL1L-HRD1 complex, with SEL1L and HRD1 being described as key players in ERAD [31,36,45]. Similarly, some proteins maintaining ER homeostasis are also subjected to continuous turnover by the ERAD pathway, as previously reported for IRE1α and ATF6 [41,42]. The hypothesis that ERAD is dynamic with auto-regulatory function has also been recently proposed, thus strengthening our observations that EDEM1 could form auto-regulatory complexes along with OS-9, SEL1L, and HRD1 [34,57]. 

Further, we analysed the association of EDEM1 and its IDR-lacking mutant with several ERAD substrates, since our previous results showed this region was essential for EDEM1–tyrosinase interaction [19]. Our results indicate the intrinsically disordered region of EDEM1 mediates protein–protein interactions with ERAD clients, and the deletion of this domain does not abolish, but does significantly reduce the association with α-1AT, NHK, BACE-476, and Ri-332. A reduced binding of ∆-EDEM1 to ERAD clients was correlated with a less efficient degradation induced by the overexpression of ∆-EDEM1 compared to EDEM1, thus confirming our previous report that weak binding of the substrate leads to a lower efficiency in degradation. Moreover, we also confirmed these results using an EDEM1-deficient cell line, which clearly confirmed the dependence of misfolded protein degradation on the presence of EDEM1-IDR. We do not exclude that the mannosidase-like domain may also bind the substrates, as previously reported [58]; however, the IDR of EDEM1 ensures the specificity and efficiency of degradation.

Considering our results showed that the IDR of EDEM1 is important for binding ERAD substrates, we also explored the idea it might affect association with components of the ERAD pathway. We found—using mass spectrometry, immunoprecipitation, and Western blotting—that some interactions with ERAD components are reduced for ∆-EDEM1 (e.g., SEL1L, OS-9, ERLEC1/XTP3-B, GRP94/HSP90B1, and HRD1/SYVN1), while others are either not-affected or even consolidated (e.g., DNAJC10, CCDC47, ANKZF1, ERLIN1, and ERLIN2). This might be explained by the less efficient association of ∆-EDEM1 with ERAD substrates, which suggests a reduced activity of ∆-EDEM1 in protein degradation and, implicitly, a lower association with partner proteins from ERAD or that it associates with selected proteins to increase its stability. 

With all these results in hand, we were next interested to know whether EDEM1 activity specifically requires partner proteins from ERAD, since, as described above, it forms functional complexes with other ERAD proteins. To our surprise, we found that the overexpression of EDEM1, and not ∆-EDEM1, was able to accelerate the degradation of ERAD substrates when either SEL1L, HRD1, OS-9, or XTP3-B were silenced, suggesting EDEM1 most likely targets ERAD substrates for degradation, independent of the SEL1L-HRD1 complex in this case. Furthermore, we discovered that EDEM1 was able to accelerate substrate degradation even when proteasomal degradation was severely impaired by silencing of p97/VCP. Therefore, we hypothesised that EDEM1-induced degradation, when ERAD is impaired, may occur via autophagy, the alternative degradation pathway in eukaryotes. 

To test this hypothesis, we used differential fractionation and immunoprecipitation to investigate whether EDEM1 has functional connections with the autophagy machinery. We found several of the proteins involved in autophagy to either co-fractionate or co-precipitate with EDEM1 and ∆-EDEM1. Our results show that EDEM1, and ∆-EDEM1 with less efficiency, induced the formation of protein aggregates, likely due to the presence of its IDR, which has the capacity to drive self-assembly, as extensively reported for other systems [53,54,55]. Furthermore, we identified three of the six reported ER-phagy receptors as potential interactors of EDEM1 that could mediate the degradation of EDEM1-induced aggregates by recruiting the autophagy cytosolic machinery [47,48,49,50,51,52]. All these results allowed us to speculate and propose that EDEM1 functions in dynamic ERAD complexes; however, in the absence of a functional ERAD or efficient proteasomal degradation, EDEM1 overexpression leads to the efficient formation of protein aggregates that are recognised by the autophagy pathway, thus restoring ER homeostasis.

In conclusion, we identified EDEM1 as part of auto-regulatory complexes in ERAD and the crucial role of the N-terminal IDR for substrate binding and accelerating their degradation, as well as productive association with some ERAD components. We also showed that EDEM1 overexpression bypasses the requirement for proteasomal degradation by driving the formation of amyloid-like oligomers and most likely recruiting the cytosolic autophagy machinery to degrade these aggregates, via association with ER-phagy receptors.

## 4. Materials and Methods

Reagents, antibodies, and plasmids: pcDNA3.1-α-1AT-HA and pcDNA3.1-NHK-HA, and pcDNA3.1-BACE-476 were a kind gift of M. Molinari (IRB, Bellinzona, Switzerland), pCI-neo-Ri-332 was generated in the lab of N.E. Ivessa, and all other plasmids were described previously [19].

Rabbit α-EDEM1 (E84060-Sigma-Aldrich, St. Louis, MO, USA) was used for Western blotting, and goat α-EDEM1 (sc-27891) was used for immunoprecipitation; goat α-SEL1L (sc-48081), MAN1B1 (sc-393145), BiP (sc-166490), GAPDH (sc-81545) were from Santa Cruz Biotechnology (Dallas, TX, USA); rabbit α-BACE1 (ab2077), rabbit α-calnexin (ab22595), rabbit α-OS-9 (ab19853), rabbit α-XTP3-B (ab181166), rabbit α-tubulin (ab18251), and rabbit α-Grp94 (ab3674) were from Abcam (Cambridge, UK); mouse α-LC3 (0231-Nanotools, Teningen, Germany) and rabbit α-1AT (A0012) was from Dako (Jena, Germany); rabbit α-ATG5 (12994) and rabbit α-HRD1 (14773) were from Cell Signaling (Leiden, Netherlands); and mouse α-actin (612657-BD Biosciences, San Jose, CA, USA) and rabbit α-Ribophorin I antibodies were described previously [59]. All siRNAs were from Santa Cruz Biotechnology, as follows: siRNA SEL1L (sc-61514), siRNA OS-9 (sc-96230), siRNA XTP3-B (sc-94979), siRNA HRD1 (sc-76620)., siRNA VCP (sc-37187), MG132 (sc-201270), and kifunensine (sc-201364). All other chemicals were from Santa Cruz Biotechnology (Dallas, TX, USA) unless specified. 

Cell culture and transfection: HEK293T, HEK293T-KO, and HeLa cells were cultivated in DMEM (cat: 10566-032) supplemented with 10% FBS (cat: 10270-098) from Gibco (Life Technologies, Paisley, UK). 24 h post-seeding, the cells were transfected using Lipofectamine 2000 (Invitrogen-Life Technologies, Paisley, UK) or polyethylenimine (PEI) (Sigma-Aldrich St. Louis, MO, USA,) (2:1 *v/w* ratio transfection reagent:DNA), according to manufacturer’s protocol, and they were harvested after 48 h. For siRNA transfection, Lipofectamine RNAiMAX (Invitrogen-Life Technologies, Paisley, UK) was used as the transfection reagent (1:1 *v/v* ratio transfection reagent:siRNA), and the cells were harvested after 72 h.

CRISPR/Cas9 generation of EDEM1-KO cell line: HEK293T cells were co-transfected with CRISPR/Cas9 KO-specific plasmids from Santa Cruz Biotechnology (Dallas, TX, USA: EDEM CRISPR/Cas9 KO Plasmid (h) (sc-401946) and EDEM HDR Plasmid (h) (sc-401946-HDR), according to manufacturer’s instruction. At 48 h post transfection, the media were changed with fresh DMEM supplemented with 4 µg/mL puromycin, a selection antibiotic. The cells were kept in the media supplemented with puromycin for 3 passages, after which they were transferred onto media supplemented with 2 µg/mL puromycin. Selection efficiency was verified by expression level of EDEM1 in normal versus KO EDEM1 cell line assessed by Western blot. For a homogenous expression, the cells were cloned using a FACS Aria III system (BD Biosciences, San Jose, CA, USA). The expression level of EDEM1 in clones was verified also by Western blotting. The clone selected for further work was supplementary tested by immunoprecipitation with EDEM1 specific antibodies followed by Western blotting detection.

Sucrose gradient fractionation: HEK293T cells transfected with EDEM1 and ∆-EDEM1, treated or not with kifunensine, were lysed either in a TritonX-100-containing buffer (1% Triton X-100 (*v/v*), 150 mM NaCl, 1.5 mM MgCl2, and 1 mM EDTA) or a Digitonin-containing buffer (1% Digitonin (*w/v*) 50 mM Tris-HCl pH 7.3, 5 mM EDTA, and 150 mM NaCl) supplemented with protease inhibitors (Roche-Basel, Switzerland), and the lysates that were cleared at 14,000 g for 30 min were loaded on a continuous 10–40% sucrose gradient, prepared in an 8× diluted lysis buffer. The samples were centrifuged in an SW41 Ti rotor (Beckman, Brea, CA, USA) for 16 h, 39,000 rpm, at 4 °C. The collected fractions were precipitated with a 100% TCA (Sigma-Aldrich, St. Louis, MO, USA) solution, 1:4 ratio, centrifuged at 4 °C, and washed 3 times with cold acetone. Dried pellets were resuspended in a 4% SDS-containing buffer (100 mM TRIS-HCl, pH: 7.60) and sonicated, and equal volumes from each fraction were separated by SDS-PAGE in reduced conditions. The proteins were transferred onto nitrocellulose membranes and probed with specific antibodies.

Inhibitors treatment: The cells were seeded in 12 well plates, transfected as described above, and incubated with 12.5 µM MG132 or 30 µM kifunensine for approximately 16 h before harvesting; for pulse-chase experiments, MG132 was added in the starvation, pulse, and chase period at a 20 μM concentration.

Western blotting: HEK293T was co-transfected with EDEM1 mutants or/and ERAD substrates, as described above. Cells were lysed in buffers containing either 1% Triton-X100, 2% CHAPS, or 1% Digitonin for 30 min on ice and centrifuged at 14,000 rpm for 30 min. Equal amounts of proteins from each sample, detected by bicinchoninic acid method (BCA), were separated by SDS-PAGE and transferred onto nitrocellulose membranes. The membranes were probed with appropriate primary antibodies for 2 h and diluted in 5% milk or BSA in phosphate buffered saline (PBS)-0.1% Tween at room temperature (RT) or ON at 4 °C, and they were washed and incubated with secondary antibodies coupled with HRP for 1h at RT. The results were viewed by chemiluminescent reaction. 

Pulse-chase and immunoprecipitation: The cells were starved in the cysteine/methionine-free medium (Sigma-Aldrich, St. Louis, MO, USA) for 30 min, pulse-labelled for 20 or 30 min with 50–75 mCi of [^35^S]-methionine/cysteine (Tran^35^S-label, Perkin Elmer, Waltham, MA, USA), and chased for the indicated time points; for some of the samples MG132 20 µM was added. Labelled cells were washed with ice cold PBS and lysed with a CHAPS buffer (50 mM HEPES, 200 mM NaCl, and 2% CHAPS). The lysates were incubated overnight with antibodies for Ribophorin I, EDEM1, or α-1AT, immobilized on protein A-Sepharose beads for 2 h at 4 °C, and eluted with a Laemmli buffer, 5× stock diluted to 1× with TE (50 mM Tris, 150 mM NaCl, and 1 mM EDTA) at 95 °C for 5 min. The samples were separated by SDS-PAGE, and proteins were visualized by autoradiography.

Immunoprecipitation and Western blotting: HEK293T or HEK293T-KO EDEM1, previously transfected with EDEM1 mutants and/or ERAD substrates treated or not with kifunensine, were harvested, lysed in a CHAPS-containing buffer and subjected to immunoprecipitation with specific antibodies, as indicated in each figure panel ON and captured on protein A/G-Sepharose beads for 2 h at 4 °C. The proteins were eluted with a Laemmli buffer, separated in polyacrylamide gels, transferred onto nitrocellulose membranes, and probed with specific antibodies. Results were visualised by chemiluminescent reaction.

Immunoprecipitation of sucrose gradient fractions: Sucrose gradient fractions of HEK293T-overexpressing EDEM1 and digitonin lysates were concatenated to have 8 out of 15 fractions initially harvested. Each fraction was diluted 10 times with a 0.1% Digitonin buffer and incubated ON with polyclonal anti EDEM1 antibodies (1:1000 *v/v* dilution). The antibodies were captured on protein A-Sepharose beads for 2 h at 4 °C. After 3 washes with a 0.1% Digitonin-containing buffer, the proteins were eluted with a Laemmli buffer and separated in polyacrylamide gels. Furthermore, the gel was cut in small pieces that were subject to trypsin digestion.

Immunofluorescence microscopy and co-localization analysis: HeLa cells were seeded onto coverslips and transfected with the corresponding plasmids for 24 h using Lipofectamine 2000 (Invitrogen-Life Technologies, Paisley, UK) according to the manufacturer’s instructions. Afterwards, the cells were fixed by incubation with 1% PFA in PBS (13 mM NaCl, 2.7 mM KCl, 10 mM Na2HPO4, and 1.8 mM KH2PO4) for 1 h and permeabilised for 3 min with 0.005% Digitonin in a blocking buffer (2% horse serum in PBS). The samples were incubated with a blocking buffer for 2 h and overnight with the primary antibodies at the indicated dilutions in humidified atmosphere. The next day, the coverslips were washed and incubated with a combination of secondary antibodies and fluorescent dye (Proteostat-Enzo Life Sciences, Farmingdale, NY, USA) to detect aggregates for 30 min at RT. Samples were extensively washed and subsequently mounted on glass slides. Images were acquired using the Zeiss LSM 700 (63X, 1.4 NA, oil) microscope using the LSM acquisition software (Zeiss, Oberkochen, Gemany).

Acquired images were processed using the ImageJ software. Co-localization analysis was performed using the ImageJ JACoP plugin (NIH, Bethesda, MD, USA). The images were split into separated channels and used for threshold processing. The analysis was performed in three independent experiments, and the total number of fields analysed is indicated in the figure legends. 

LC–MS/MS analysis: HEK293T cells expressing an empty vector, EDEM1, or ∆-EDEM1 were harvested at 90% confluence and lysed using either 1% Triton X-100 (TX)- or 1% Digitonin (DIG)-containing buffers for 30 min on ice. The lysates were cleared by centrifugation, followed by immunoprecipitation using polyclonal anti-EDEM1 antibodies, as previously described [19]. The captured complexes were eluted from the resin with a soft elution buffer (SEB: 50 mM Tris pH 8.0, 0.2% SDS, and 0.1% Tween-20) in a 4:1 (*v/v*) ratio at RT [60]. The eluted proteins were separated by SDS-PAGE and prepared for MS analysis using a previously described protocol for in-gel digestion [61]. For HEK293T cell proteomic analysis, proteins were extracted using 6M Guanidine hydrochloride, reduced with 10 mM TCEP (Tris(2-carboxyethyl)phosphine hydrochloride), alkylated with 5 mM chloroacetamide, and subjected to in solution overnight digestion with trypsin at 37 °C. The extracted peptides were dried in Speed-Vac, and each sample was reconstituted in mobile phase A (0.1% FA and 2% ACN) and injected on a C18 trap column (20 mm × 100 µm internal diameter) (Proxeon Biosystems, Thermo Scientific, Waltham, MA, USA) connected online to a C18 analytical column (100 mm x 75 µm internal diameter) (Proxeon Biosystems, Thermo Scientific, Waltham, MA, USA) for peptide separation. The chromatographic equipment was connected online to an LTQ-Orbitrap Velos Pro instrument operated in a data-dependent mode. A top 5, 10, or 15 method, depending on sample complexity, was used for data acquisition involving a survey scan at 60,000 resolution (m/z 400) with Orbitrap detection, followed by the consecutive collision-induced dissociation (CID) fragmentation scans in the linear ion trap. A 2–30% B (0.1% FA and 98% ACN) gradient was used for the chromatographic separation of the peptides. 

LC–MS/MS data analysis: Raw data files were searched using the SEQUEST/SEQUESTHT algorithms integrated into Proteome Discoverer v1.4 (Thermo Scientific, Waltham, MA, USA) or using the Andromeda integrated in MaxQuant. For both searches, the settings were the following: trypsin as the proteolytic enzyme and a maximum of two missed cleavages, 10 ppm as mass accuracy for precursor ions or 20 ppm (during the first search) and 6 ppm (for the second search) for Andromeda searches, 0.5 Da for fragment ion tolerance, carbamidomethylation on Cys residues as a static modification, and oxidation on Met residues as a dynamic modification. For PSM validation, the percolator node available in Proteome Discoverer v1.4 was used, with the validation based on q value. The results were filtered for 1% FDR (PSM level) and a peptide mass deviation of maximum 5 ppm. Andromeda results were similarly filtered to 1% FDR using the built-in MaxQuant procedure

Statistical analysis: For statistical analysis, data sets were processed using either one-way or two- way ANOVA with a Bonferroni correction using Prism6 (GraphPad software, San Diego, CA, USA). Results with a *p* value of less than 0.05 were considered significant, as indicated in the figure legends. No criteria of inclusion or exclusion of data were used in this study. Data shown are representative for two-to-four experiments, as specified in the figure legends. 

For LC–MS/MS data analysis, a two-sample *t*-test was used with a permutation-based FDR correction at a significance value of 0.05 using the MaxQuant reported intensity values (LFQs). Shown in figures are either log of LFQ or spectral counts for the SEQUEST/ SEQUESTHT searches.

The mass spectrometry proteomics data have been deposited to the ProteomeXchange Consortium via the PRIDE partner repository, http://proteomecentral.proteomexchange.org/cgi/GetDataset?ID=PXD019066 with the dataset identifier PXD019066.

## Abbreviations

ERAD	Endoplasmic reticulum-associated degradation
LC–MS/MS	Liquid chromatography–mass spectrometry/mass spectrometry
IDR	Intrinsically disordered region
LFQ	Label-free quantification
PSM	Peptide-spectrum match
α-1AT	α1-antitrypsin
NHK	Null Hong Kong variant of α1-antitrypsin
Ri	Ribophorin I
BACE	Beta-secretase
E1-KO	EDEM1 knock-out
∆-EDEM1	EDEM1 mutant lacking the IDR
CNX	calnexin
UPR	Unfolded protein response
